# Statistical analysis of immuno-functionalized tumor-cell behaviors on nanopatterned substrates

**DOI:** 10.1186/1556-276X-7-637

**Published:** 2012-11-22

**Authors:** Dong-Joo Kim, Geehee Lee, Gil-Sung Kim, Sang-Kwon Lee

**Affiliations:** 1 Basic Research Laboratory (BRL), Department of Semiconductor Science and Technology, Chonbuk National University, Jeonju, 561-756, South Korea; 2 Department of Biology, Emory University, Atlanta, GA, 30322, USA

**Keywords:** Nanowire arrays, Cell adhesion, Circulating tumor cells, Filopodia, Cell migration, Cell capture efficiency

## Abstract

Laser scanning cytometry has been proven as a powerful technology for high-content, high-throughput quantitative analysis of cellular functions in a fully automated manner. It utilizes a large-area fluorescence imaging scheme and rigorous image quantitation algorithms to enable informative analysis of cell samples attached to solid substrates. While this technology represents a powerful approach for high-content screening using cell lines, it has not been applied to the study of tumor-cell behaviors on these solid nanopatterned substrates after several hours of incubation. Herein, we statistically demonstrated functional cellular morphology information, including size, shape, and distribution of the captured cells after 0.5 to 45 h of incubation on nanopatterned substrates, such as silicon nanowires and quartz nanopillars, along with planar glass substrates. With increasing incubation time up to 45 h, we observed that the nanopatterned substrates could have not only increased adhesion and traction forces between cells and nanopatterned substrates, but also limited cell spreading on the substrates compared to the planar glass substrates. On the basis of our results, we suggest that the most important factors to influence the cell behaviors on the three solid substrates are the degree of dimension on cell behaviors and cell traction force.

## Background

Nanostructures have been increasingly used for studies on cell interaction with solid nanostructures because unique properties of nanostructured surfaces enable a variety of novel functions of immobilized cells on the nanostructured surfaces [1-3]. For example, Qi et al. recently reported that nanometer-scale topography influences diverse cell behaviors, including cell adhesion, motility, proliferation, and differentiation [4]. In addition, owing to high surface area and increased nanowire-cell surface interaction, nanowire arrays functionalized with capture agents were also demonstrated for high-yield capture of surface-bound cells including immune cell subsets and circulating tumor cells (CTCs) [5-7]. We have also demonstrated a novel platform for separating CD4^+^ T lymphocytes from mouse splenocytes using streptavidin (STR)-functionalized and vapor–liquid-solid-grown silicon nanowire (SiNW) [5] as well as transparent quartz nanopillar (QNP) arrays [8] having a higher separation efficiency of 93% to 95.3%. More recently, we reported on the development of nanowire substrate-enabled laser scanning cytometry (LSC) for cell analysis in order to achieve quantitative, automated, and functional evaluation of the circulating tumor cells where the captured rare cells were at the very early stage of incubation (<0.5 h) [6]. In a previous study, we clearly demonstrated that the LSC method enables large-area, automated quantitation of captured cells and rapid evaluation of functional cellular parameters (e.g., size, shape, and protein levels) at the single-cell level [6]. In addition, more detailed studies are still required on how the various size and shape-matched nanostructures and nanomaterials interact in the targeted cells at different times of incubation (>1 h) using pre-proven LSC technique.

Herein, we demonstrate microarray scanner-based imaging cytometry representing a high-content, high-throughput approach to statically characterize tumor cell captured behaviors on nanostructured microchips after 1 to 48 h of incubation on two sets of nanostructures: QNP and SiNW substrates.

## Methods

**Figure 1:**
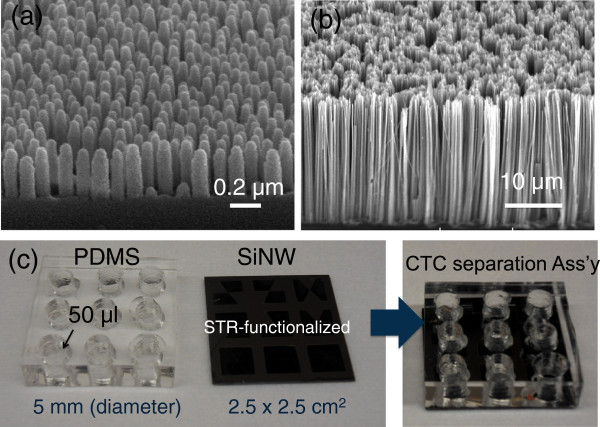
**Tilted SEM images.** (**a**) QNP arrays and (**b**) wet chemically etched SiNW arrays. (**c**) Photographic images of artificial CTCs capturing/isolating assembly including nine cell-separation chamber (PDMS) and wet-chemically etched SiNW arrays (2.5 × 2.5 cm^2^). Both surfaces of QNP and SiNW arrays were functionalized by a high affinity biotin-STR conjugation technique.

QNP arrays (2.5 × 2.5 cm^2^) were fabricated by a series of processes including colloidal-polystyrene (PS)-nanoparticle (NP) coating and templating, metallization for pattern transfer, and reactive ion etching (RIE) for constructing nanopillar structures [6]. The PS NP monolayer was first coated onto a quartz substrate using a modified self-assembly technique we recently developed [6]. Oxygen plasma etching was then performed for 5 s with a mixed gas of O_2_/Ar and a RF power of 100 W for securing space between the PS NPs on the quartz substrate. Evaporated Cr metal of approximately 25-nm thickness was then lifted off with the solution of *N*-methyl-2-pyrrolidone in ultrasonic bath. In producing the QNP arrays, RIE process was performed for 4 min with a mixture gas of SF_6_/Ar, a pressure of 20 mTorr, RF power of 300 W, and bias power of 100 W. For the QNP RIE process, Ni metal of approximately 30 nm served as an etching mask. The remaining Ni metal was completely removed with nickel etchant (LCE-12 K, Cyantek Corporation, CA, USA). Figure 1a shows the tilted scanning electron microscope (SEM) image of as-prepared QNP arrays.

Next, SiNW arrays (2.5 × 2.5 cm^2^) were prepared by Ag-assisted chemical wet-etching process of p-type Si wafer immersed into 10 wt% hydrofluoric (HF) acid for 5 min to remove the native oxide layer and sequentially treated in a boiled RCA solution (H_2_O_2_/NH_4_OH/H_2_O = 1:1:5) for 1 h to create a hydrophilic surface. An Ag film with a thickness of 30 nm was coated onto (100) Si substrates with the resistivity of 1 to 10 Ω·cm, which were cleaned by electroless deposition in an aqueous solution [9]. The cleaned Si samples were placed in 10% HF and 5 × 10^−3^ M AgNO_3_ solution at room temperature for 5 min. The Ag-coated Si samples were then immersed in an aqueous solution containing 10% HF and 0.3% H_2_O_2_ at room temperature for 30 min. The Ag metals remaining on the Si substrates were removed in boiling aqua regia (HCl/HNO_3_ = 3:1) for 1 h and by additional amorphous Si etching for 30 s in buffered oxide etchant (NH_4_F/HF = 6:1). Figure 1b shows the SEM images of wet chemically etched SiNW arrays with tilted view.

Prior to the surface functionalization, three as-prepared nanopatterned substrates (QNP, SiNW, and planar glass substrates for control samples) were carefully cleaned with H_2_O_2_/H_2_SO_4_ (1:1) for 10 min to remove all of the organic materials and impurities on the surface. Then, the substrates were washed using a conventional three-step cleaning process (acetone, isopropyl alcohol, and DI water) and dried with air. The surface was treated with O_2_ plasma for 20 s to confer the hydroxyl groups on the substrate surfaces. The surface of the two-nanopatterned substrates with planar glass substrates was functionalized by STR-immobilization method we developed previously (Figure 1c) [5]. During this procedure, we first applied (3-aminopropyl)-triethoxysilane to aminate the nanowire surface, which can be further functionalized with STR via a two-step aldehyde/amine reaction using glutaraldehyde as the linker. Finally, biotinylated anti-human monoclonal anti-human epithelial cell adhesion molecule (EpCAM), where the targeted cells were pre-mixed, was introduced to the STR-functionalized nanowires through the high-affinity biotin-STR binding. Cell-capture chambers with nine circular wells (5 mm in diameter, Figure 1c) were made by molding a polydimethylsiloxane (PDMS) elastomer. The solidified PDMS mold was cut to the size of 2.5 × 2.5 cm^2^, which is the same size of the surface-functionalized nanopatterned substrates. A solution of the A549 cells (human lung carcinoma cell line, CCL-185) purchased from American Type Culture Collection (ATCC, VA, USA), which are conjugated with biotin-EpCAM-Abs in F12K:DMEM (500 mL, Invitrogen Corporation, NY, USA), with a final volume of approximately 50 μL was then pipetted into each of the nine cell-capture chambers with cell populations of approximately 10^3^ cells/chamber. Three samples with nine cell-capture chambers were prepared in each group (0.5, 21, and 45 h), and the average spreading areas and sizes of each fixed cell on three different substrates were consequently calculated with standard deviation (*n* = 9).

**Figure 2:**
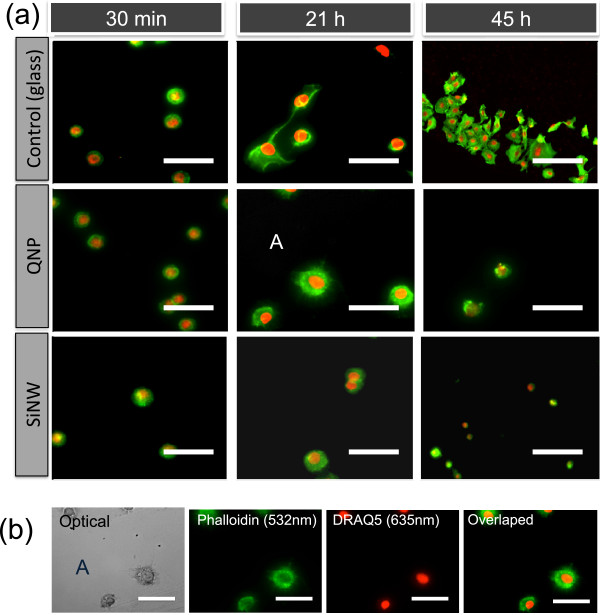
**Optical and fluorescence images of A549 cells.** (**a**) Flourescence images of A549 cells cultured on three different substrates (planar glass, QNP, and SiNW arrays) at 37°C for 0.5, 21, and 45 h. (**b**) Optical and fluorescence images of the immobilized A549 cells on STR-functionalized QNP arrays after 21 h of incubation at 37°C (marked area A in the second row of (a)). Before fluorescent imaging, the actin filaments were stained with Alexa Fluor 594 conjugated phalloidin (Invitrogen, green 532 nm) for 20 min, and the nucleus was stained with DRAQ-5 (Cell Signaling, red 635 nm).

For straightforward detection of the captured A549 cells, a standard immunofluorescent staining procedure was\ performed on cells fixed on STR-functionalized SiNW, QNP, and planar glass substrates. The PDMS cell-capture chambers were first washed out with 1 × PBS and Tween-20 (PBST, KPL Inc., USA) at least three times to remove unbound tumor cells on the SiNW arrays, and then fixed with 4% paraformaldehyde for 15 min. The cells were immersed in blocking buffer (5% bovine serum albumin and 0.3% Triton X-100 in 1 × PBS) for 60 min. After rinsing with PBS three times for at least 5 min each, the actin filaments and nuclei were stained with Alexa Fluor 594 conjugated phalloidin (Invitrogen, green 532 nm) for 20 min and with DRAQ5 (Cell Signaling Technology, Inc., MA, USA, red 632 nm) for 5 min (Figure 2). The samples with cultured cells were then given a three-step cleaning process, using PBS, PBS in DI water (1:1), and DI water after peel-off of PDMS cell-capture chambers. The samples were finally transferred to a microarray scanner for further LSC analysis.

For the image of surface-bound cells on the nanopatterned substrates, an Axon Genepix microarray scanner 4000B (Molecular Devices, LLC, CA, USA) was used. Green and red YAG lasers (532 and 635 nm) were used to visualize the captured cells (actin, green 532 nm) and nuclei (red 635 nm) on the three different STR-functionalized substrates with approximately 5-μm resolution. The cell-capture platform was automatically scanned, and the scanned images of PDMS cell-capture chambers that contained the captured cells were transported into CellProfiler^TM^ ( http://www.cellprofiler.org) cell image software for rapidly quantitation of the captured cells on STR-functionalized dual-nanopatterned substrates with planar glass substrates.

## Results and discussion

**Figure 3:**
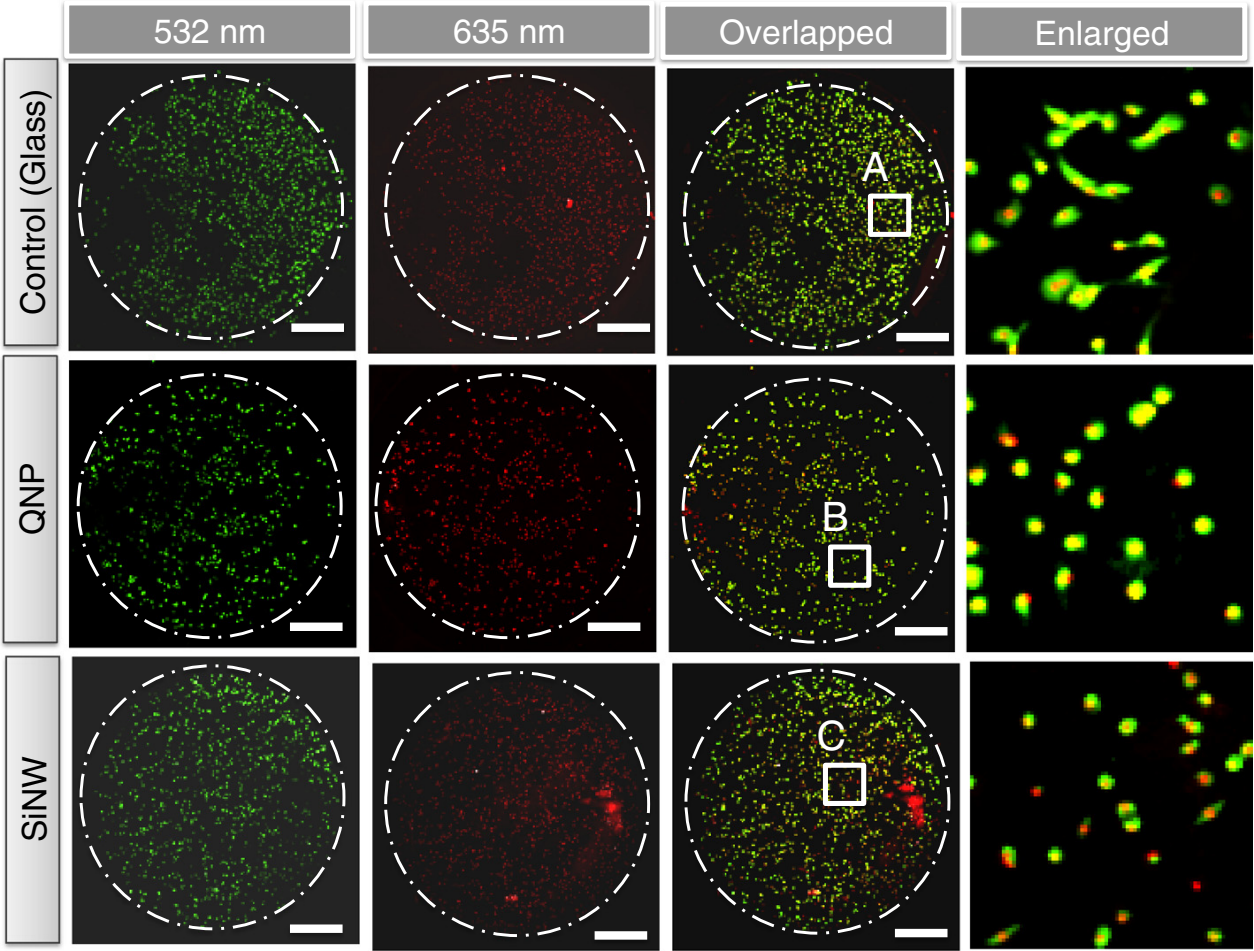
**Laser-scanned fluorescence images of A549 cells on three different substrates after 21 h of incubation at 37°C.** First and second columns show low magnification of actin (phalloidin-stained 532 nm) and nucleus (DRAQ-5-stained 635 nm) of immobilized A549 cells on the three different substrates, respectively. Third column shows the overlapped images of immobilized A549 cells on STR-functionalized nanostructured substrates as well as planar glass substrate (as a control). Fourth column shows the enlarged images of immobilized A549 cells on marked areas **A**, **B**, and **C** in the third column, respectively.

**Figure 4:**
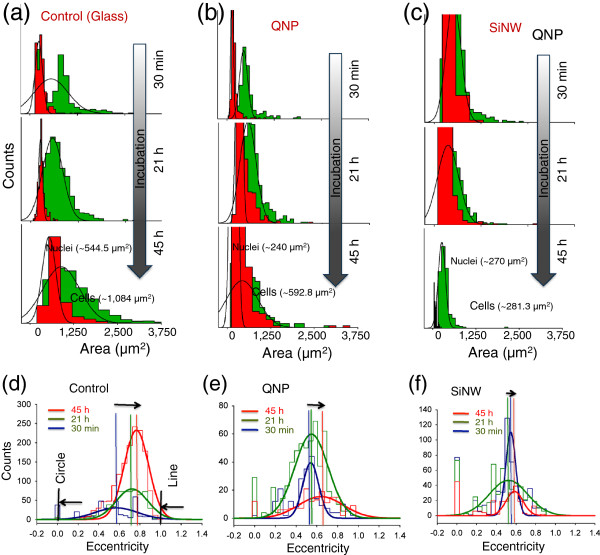
**The size histogram and the eccentricity of A549 on the three different substrates.** (**a**) to (**c**) The size histogram (area in μm^2^) and (**d**) to (**f**) the eccentricity, which are produced by CellProfilerx^TM^, of immobilized A549 cells (phalloidin-stained 532 nm) on planar glass, QNP arrays, and SiNW arrays, respectively, using laser microarray scanner (both green 532 nm and red 635 nm) after 0.5 to 45 h of incubation at 37°C. The eccentricity is defined by the ratio of the distance between the foci of the ellipse and its major axis length.

**Figure 5:**
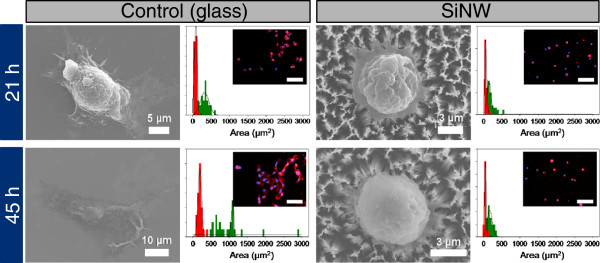
Size distribution and adhesion of SH-J1 on glass (flat substrate) and SiNW for 21 and 45 h of incubation.

**Figure 6:**
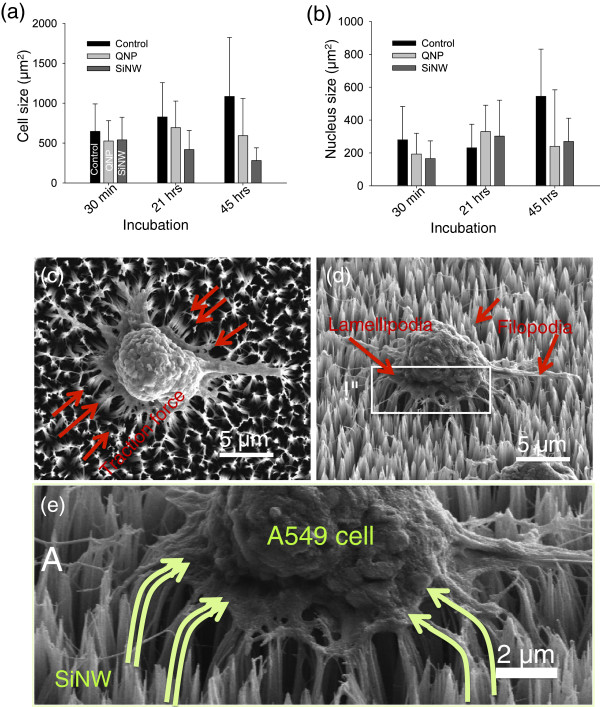
**Size distribution and enlarged SEM images of A549 cells.** (**a**) Cell size and (**b**) nucleus size distribution (in μm^2^) of immobilized A549 cells on STR-functionalized three different substrates (planar glasses, QNP arrays, and SiNW arrays) after 0.5 to 45 h of incubation. (**c**) to (**e**) Top, tilled, and enlarged SEM images of A549 cells bound on SiNW arrays after 21 h of incubation at 37°C. The SiNW substrate was bent toward the center of the cells due to the high traction force these cells exerted (see arrows which indicate the bending direction).

For the cell adhesion and migration study, two types of nanostructures (QNP and SiNWs), along with planar glass substrates, were used. The diameter and length of QNP arrays are ranging from 100 to 140 nm and from 300 to 500 nm, respectively, while SiNWs have dimensions of 10 to 100 nm in diameter and 25 to 30 μm in lengths as shown in Figure 1a,b. The A549 cells were reacted with biotinylated EpCAM antibody (anti-human CD326-Ab, eBioscience, Inc. CA, USA) and then reacted at 4°C for 20 min on STR-functionalized substrates (SiNW, QNP, and planar glass) prior to loading the A549 cells, which were manually counted using a hemocytometer with approximately 10% error (Hausser Scientific Co., PA, USA), into the PDMS cell-capture chambers (Figure 1c). The A549 cells were cultured on three types of substrates for 0.5, 21, and 45 h at 37°C. Then, future cellomic parameters (spreading area (size) of the cells and nuclei, eccentricity, etc.) were characterized using further immunofluorescence and the microarray scanner-based high-content imaging technique we developed previously [6,8]. Figure 2 shows the overlapped fluorescence images of A549 cells (actin with green 532 nm, nucleus with red 635 nm) captured on three different types of substrates at 37°C for 0.5, 21, and 45 h of incubation. At 0.5 h of incubation, the A549 cells immobilized on the three types of substrates display fundamentally similar morphology and spreading area, as shown in Figure 2a (first column). With an increase of incubation time to 21 h, the cells on the two types of nanostructures (QNP and SiNWs) have different morphology compared to those on planar glass substrates (second column in Figure 2a), indicating that the cells on nanostructures (QNP and SiNWs) restricted themselves to a smaller surface spreading area. With further increasing incubation time to 45 h, the A549 cells cultured on planar glass substrates were well spread over the substrates, while the spreading behaviors of the cells on the two types of nanostructures were completely limited in the spreading area as indicated in Figure 2a (third column). From fluorescence microscopy, we noticed that the fluorescence images provided very limited information on cell morphology to statically qualify the cell development on the three types of substrates. On the other hand, we realized that LSC method provided a powerful technology for high-content, high-throughput quantitative analysis of cellular functions, as we reported previously [6]. Figure 3 shows microarray-scanned fluorescence images of A549 cells cultured on three substrates after 21 h of incubation. As shown in Figure 3, the cell morphology, including nuclei on the three types of STR-functionalized substrates, were visualized by a green and red YAG laser-based microarray scanner and transferred to CellProfiler^TM^ cell image software for further statistical analysis of the cell morphology. Figure 4 shows the size histograms and eccentricity, which is defined by the ratio of distance between the foci and the ellipse and its major axis length, of immobilized A549 cells on three substrates for incubation of 0.5, 21, and 45 h using a microarray scanner. To further understand the effect of cell spreading limitation on other cell line, we also cultured SH-J1 (sarcomatoid hepatocellular carcinoma cell line) on planar glass substrate and Si NW for 21 h and 45 h in incubator (5% CO_2_, 37°C) [10], and statically demonstrated cell morphology such as size, adhesion, and migration. As shown in Figure 5, the size of SH-J1 on planar glass has increased with incubation time and freely migrated on the glass substrate, whereas the SH-J1 cells on SiNW substrate have a spreading limitation due to SiNW bending, which was caused by interaction with a cell traction force and a stiffness of SiNWs. These cell behaviors on planar glass and SiNWs substrates were nearly identical to those of A549 cell line. Figure 6a,b shows the summary of the cell and nucleus size distribution of immobilized A549 cells on the three STR-functionalized substrates shown in Figure 4a,b,c. The histograms (Figure 4a,b,c) show that the spreading of lung cancer cells (A549) on vertical SiNWs and QNP substrate is significantly decreased compared to that on a planar glass substrate (Figure 4a and Figure 6a,b), with an increase of incubation time up 45 h. The average spreading area for SiNW arrays was reduced from 540.0 ± 283.3 to 281.3 ± 158.8 μm^2^, while the spreading area for QNP arrays showed no significant changes in size, ranging from 525.8 ± 254.8 to 592.8 ± 466.3 μm^2^. At incubation of 45 h (Figure 4c and Figure 6a,b), the spreading area on SiNW arrays (281.3 ± 158.8 μm^2^) was three to four times smaller than that on planar glass substrates (approximately 1,084.88 μm^2^), indicating the immobilized A549 cells on SiNWs restricted themselves to a much smaller area and were not well-cultured on the nanostructured substrates. This result is similar to those we observed previously in the fluorescence microscopic images shown in Figure 2. These results suggest the critical role of nanostructures in cell adhesion, spreading, and likely migration, compared to the planar glass substrates. We also suggest that the restriction of cell migration on SiNWs after several hours of incubation results in three-dimensional (3-D) movement of the A549 cells on SiNWs, while the cells on planar glass show only 2-D migration behaviors. As indicated in Figure 6c,d,e, the cells on SiNW arrays were gripping the NWs around and underneath with protruding filopodia or lamellipodia, presenting 3-D migration on SiNWs. Consequently, the NWs were bending to the center of the cells due to high traction force, as reported previously [11]. In contrast, cells on planar glass were freely spread (2-D migration) without the contribution of traction force between the substrates and the cells. As a result, 3-D migration of the cells unquestionably limits cell spreading of the nanostructures. Moreover, it was noticed that the average spreading area on SiNW substrates was considerably lower than that on other nanostructure (QNP) arrays with increase in incubation time, as shown in Figure 4b,c and Figure 6a,b. This difference in cell migration between the two types of nanostructures can be explained by the difference in material stiffness. Due to the relatively weak stiffness, the SiNW arrays with the cultured cells were easily bent to the center of the cells compared to the QNP substrates. Hence, the cells on SiNWs could not migrate freely over the substrates. Consequently, the spreading areas gradually became smaller than those on QNP with increasing incubation up to 45 h due to the higher traction force acting on SiNW arrays. This is in good agreement with previous reports in cell migration with an increase in culture time from 2 to 36 h [11].

To further quantify the shape of the cells immobilized on the nanostructures and to support the migration behaviors of the nanostructures, the eccentricity of the cultured cells on the substrates was calculated using CellProfiler software. Figure 4d,e,f show the summary of eccentricities of the cells for the three different substrates after 0.5 to 45 h of incubation. As we mentioned previously, the value in eccentricity is between 0 and 1. (0 and 1 are degenerate cases; an ellipse whose eccentricity is 0 is actually a circle, while an ellipse whose eccentricity is 1 is a line segment). Therefore, the average cell shapes on both SiNWs (approximately 0.44 ± 0.25) and QNP (approximately 0.58 ± 0.24) substrates were more circular compared to the planar glass substrates (approximately 0.72 ± 0.15), indicating the cells on planar glass were well spread and freely cultured on the substrates.

## Conclusion

In summary, we statistically demonstrated the functional cellular morphology parameters including size, shape, and distribution of the captured cells after 0.5 to 45 h of incubation on nanopatterned substrates of SiNW and QNP, along with planar glass substrates, using a powerful high-content LSC method. With increasing incubation time up to 45 h, we observed that the nanopatterned substrates could not only increase the adhesion and traction force between the cells and nanopatterned substrates, but also limit the cell spreading on the substrates compared to the planar glass substrates. On the basis of our results, we found that the most important factors influencing the cell behaviors on the three sets of solid substrates are the degree of dimension (2-D or 3-D migration) in cell behaviors and the cell traction force. All together, this work demonstrates the utility of microarray scanner-based high-content imaging for post-capture characterization of tumor cells captured on nanostructured microwells and the versatility of this approach for functional characterization of cell behaviors including filopodia or lamellipodia evolution with nanostructured surfaces.

## Competing interests

The authors declare that they have no competing interests.

## Authors' contributions

DJK and GSK carried out the synthesis of nanostructures including silicon nanowires and quartz nanopillars, and fluorescence measurements. DJK also prepared the samples for the SEM measurements and part of the drafted manuscript. GHL worked the fluorescence measurements and helped incubate the cells most of the time. SKL organized all the experiments and prepared most of the data and final manuscript. All authors read and approved the final manuscript.
